# Quality gap of educational services in viewpoints of students in Hormozgan University of medical sciences

**DOI:** 10.1186/1472-6920-8-34

**Published:** 2008-06-18

**Authors:** Teamur Aghamolaei, Shahram Zare

**Affiliations:** 1 Department of Health Services, School of Public Health, Hormozgan University of Medical Sciences, Bandar Abbas, Iran; 2 Department of Social Medicine, School of Medicine, Hormozgan University of Medical Sciences, Bandar Abbas, Iran

## Abstract

**Background:**

Higher education is growing fast and every day it becomes more and more exposed to globalization processes. The aim of this study was to determine the quality gap of educational services by using a modified SERVQUAL instrument among students in Hormozgan University of Medical Sciences.

**Methods:**

A cross-sectional study was carried out at Hormozgan University of Medical Sciences in 2007. In this study, a total of 300 students were selected randomly and asked to complete a questionnaire that was designed according to SERVQUAL methods. This questionnaire measured students' perceptions and expectations in five dimensions of service that consists of assurance, responsiveness, empathy, reliability and tangibles. The quality gap of educational services was determined based on differences between students' perceptions and expectations.

**Results:**

The results demonstrated that in each of the five SERVQUAL dimensions, there was a negative quality gap. The least and the most negative quality gap means were in the reliability (-0.71) and responsiveness (-1.14) dimensions respectively. Also, there were significant differences between perceptions and expectations of students in all of the five SERVQUAL dimensions (p < 0.001).

**Conclusion:**

Negative quality gaps mean students' expectations exceed their perceptions. Thus, improvements are needed across all five dimensions.

## Background

Education is a service directly impacted on by the provider. Higher education institutions are placing greater emphasis on meeting students' expectations and needs. As universities continue to become more student oriented, student perceptions of higher educational facilities and services are becoming more important [1]. Educational services quality, emphasizing student satisfaction, is a newly emerging field of concern in the medical sciences universities of Iran.

The contradictory meanings of quality education have led to the adoption of different methods for measuring quality in higher education [2]. Most of the studies focused on either measuring teaching quality or evaluating students' learning experiences [3-5].

Interest in the measurement of service quality is high, however, as highlighted by several researchers, service quality is an elusive and abstract concept that is difficult to define and measure [6-8]. For several years, academic researchers measured service quality by employing uni-dimensional scales; uni-dimensional scales, however, are inappropriate to measure a multi-dimensional concept like quality [9]. Parasuraman, Zeithmal and Berry constructed a multi-item scale measuring perceived service quality. This scale is SERVQUAL. The SERVQUAL instrument represents a multi-item scale that can be used for measuring perceptions and expectations of service quality as perceived by consumers [10]. This scale assesses customers' perceptions and expectations of service quality along five dimensions: tangibles (the appearance of the school physical facilities, equipment, personal, and communication materials), reliability (the school's ability to perform the promised services dependably and accurately), responsiveness (the school's willingness to help students and provide prompt service), assurance (the knowledge and courtesy of school office staff/faculty and their ability to convey trust and confidence), and empathy (the school office staff's and faculty's ability to provide a caring and individualized attention to students) [9].

Berry (1995) suggests that service plays an important role in enhancing value, and can positively influence a firm's success. Understanding and measuring customer expectations and performance are an essential component that can be used to enhance a company's service provision [11]. The aim of this study was to determine the quality gap of educational services by using a modified SERVQUAL instrument among students in Hormozgan University of Medical Sciences. This study helps to locate areas of performance where improvements are needed, or areas where resources could be better utilized.

Parasuraman et al., (1988) defined service quality as the gap between consumers' expectations and perceptions [10]. Gap analysis is not new in a higher educational context, and a number of studies have been influenced by the work of Parasurman et al [10]. For example, Long et al (1999) used "gap analysis" to develop a number of questions in order to compare what students "look for" (expect) and what they "experience" on a course [12]. Sander et al. (2000) meanwhile examined undergraduates' expectations and preferences in teaching, learning, and assessment [13]. LaBay and Comm (2003) also developed a number of measures to evaluate student expectations and perceptions, concerning their tutor, on a sample of undergraduate and distance learning students [14].

## Methods

The study population consisted of students in Hormozgan University of Medical Sciences in 2007. This university has three schools including a medical school, health school, and nursing and midwifery school and is located in Hormozgan province in the south of Iran. The subjects were students in general medicine, family health, disease control, environmental health, medical entomology, radiology, operation room, anesthesia, medical records, laboratory sciences, nursing and midwifery fields. A total of 300 students were selected by multi-stage sampling. Proportional to the number of students in each school, the number of students in each course and educational level, the number of subjects was determined in each group. Then in each group the subjects were selected randomly. Only the students who had studied at least one term were included in the study.

The instrument was an adaptation of the SERVQUAL survey. The original SERVQUAL survey was specifically designed to assess organizations and businesses in the service sector [10]. Some changes were made to adapt this study's survey to an academic setting. This adaptation of the SERVQUAL survey was made up of twenty-seven parallel likert scale items measuring five postulated dimensions of service quality, which consist of tangibles (4 items), reliability (7 items), responsiveness (5 items), assurance (5 items), and empathy (6 items). This questionnaire was tested in a sample of students at Zahedan University of Medical Sciences (Iran) by Kebriaei and Roudbari [15]. Its content validity and reliability was determined by them. Alpha coefficients of assurance, responsiveness, empathy, reliability and tangibles dimensions were 0.79, 0.78, 0.79, 0.89 and 0.85 respectively.

The students were first asked to rate the educational services quality (students' perceptions of current condition). To do so they were asked to select one response in each item including very good, good, moderate, poor and very poor. They were then asked to rate how important each item is to the quality of service provided (students' expectations of optimal condition). In order to do this, the students selected one response including very important, important, moderate, less important and least important. They were told that most important is equal to highest expectation and least important is equal to lowest expectation here. Each item was scored from 1 to 5 with 1 representing very poor/least important and 5 representing very good/very important. In each dimension, the scores of the items were added up and the result was divided by the number of its items. The score of perceptions and expectations of students in each dimension was from 1 to 5. The difference between perceptions (P) and expectations (E), (P-E = Q) represents the measure of service quality (Q). Where Q is negative, a service gap exists. However, where Q is positive, students' expectations are greater than their perceptions.

Descriptive statistics, paired t-test, Wilcoxon, Friedman and ANOVA were utilized to evaluate and analyze the data by SPSS13 software. The means were used to compare the students' perceptions and expectations of educational service quality and the gap between them.

This study was approved by the Medical Ethics Committee of Hormozgan University of Medical Sciences. The procedures of the study were explained to all subjects, and all provided informed consent.

## Results

The mean age of students was 21.5 ± 1.9 years.115 (38.3%) of them were male and 185 (61.7%) were female. In this university female students outnumber their male peers. 147 (49%) were in Associate degree level, 82 (27.3%) in Bachelor of Science (BS) level, and 71 (23.7%) in general medicine level. 71 (23.7%) were in medical school, 152 (50.6%) were in nursing and midwifery school and 77 (25.7%) were in health school.71 (23.7%) were students in medicine, 22 (7.3%) in family health, 17 (5.7%) in disease control, 20 (6.7%) in environmental health, 19 (6.3%) in medical entomology, 18 (6%) in radiology, 13 (4.3%) in operation room, 17 (5.7%) in anesthesia, 19 (6.3%) in medical documents, 16 (5.3%) in laboratory sciences, 34 (11.3%) in nursing, and 34 (11.3%) in midwifery fields.

The results indicated that in all five SERVQUAL dimensions, there were negative quality gaps. The least and the most negative quality gap means were in the reliability and responsiveness dimensions respectively (Table 1). There were significant differences between perceptions and expectations of students in all five SERVQUAL dimensions (p < 0.001). Also statistically there were significant differences between negative quality gaps in all five SERVQUAL dimensions (Friedman test: X^2 ^= 86.4, p < 0.001). The differences between negative quality gaps in each of the five SERVQUAL dimensions, except between assurance dimension and empathy and tangibles dimensions, were significant (p < 0.001). These dimensions, with regard to negative quality gaps, can be classified into three groups, so that the responsiveness dimension is placed in one group, the assurance, empathy and tangibles dimensions are placed in another group, and the reliability dimension is placed in a third group.

**Table 1:** Mean level of the students perceptions, expectations and service gaps in five SERVQUAL dimensions

Service Dimensions	Perceptions	Expectations	Service gaps	Paired T-Test	
				t	P
Assurance	3.23 ± 0.64	4.13 ± 0.78	-0.89 ± 0.91	-16.8	<0.001
Responsiveness	2.78 ± 0.70	3.92 ± 0.86	-1.14 ± 1.03	-18.9	<0.001
Empathy	3.07 ± 0.69	4.03 ± 0.87	-0.95 ± 0.91	-17.9	<0.001
Reliability	3.37 ± .061	4.07 ± 0.77	-0.71 ± 0.81	-15.1	<0.001
Tangibles	3.10 ± 0.79	3.94 ± 0.91	-0.84 ± 1.05	-13.9	<0.001
Total service quality	3.13 ± .054	4.03 ± 0.75	-0.89 ± 0.78	-19.6	<0.001

Also the results showed that in all of the items there were negative quality gaps (Table 2), and there were significant differences between perceptions and expectations of students in all of them (p < 0.001).

**Table 2:** Mean level of the students perceptions, expectations and service gaps in all of SERVQUAL items

Items	P*	E**	Service Gaps	Paired T-Test	
				t	P
Assurance					
1. Facilitating discussion and interaction about lessons in class	3.36	4.08	-0.72	-10.4	<0.001
2. Qualifying students for future job	2.98	4.24	-1.26	-16.4	<0.001
3. Accessibility of faculty members outside of class to Answer students' questions	3.07	3.80	-0.73	-9.40	<0.001
4. Accessibility of adequate references to increase students' professional knowledge	3.38	4.20	-0.81	-11.1	<0.001
5. Faculty members professional knowledge adequacy	3.39	4.32	-0.93	-13.5	<0.001
					
Responsiveness					
6. Supervisors accessibility when students need them	3.00	4.03	-1.03	-11.7	<0.001
7. Easy accessibility of administrators for students to express views about the curriculum	2.45	3.86	-1.41	-16.5	<0.001
8. Considering students' views and suggestions in curriculum	2.40	3.92	-1.51	-17.6	<0.001
9. Introducing suitable references to students for reading	3.38	4.08	-0.70	-9.80	<0.001
10. Declaring hours that students can refer to faculties to talk about educational problems	2.70	3.73	-1.03	-12.6	<0.001
					
Empathy					
11. Assigning suitable and relevant homework	3.10	3.64	-0.54	-7.10	<0.001
12. Faculty members flexibility when exposing to specific conditions of each student	2.77	4.04	-1.27	-15.1	<0.001
13 Convenience of class hours	2.99	4.06	-1.07	-12.8	<0.001
14. Existence of silent and convenient place in school for reading	2.98	4.03	-1.05	-12.7	<0.001
15. Respectful treatment of school staff with students	3.03	4.04	-1.00	-11.7	<0.001
16. Respectful treatment of faculty members with students	3.56	4.35	-0.79	-12.3	<0.001
					
Reliability					
17. Presenting educational content regularly and relevant	3.43	4.16	-0.72	-10.6	<0.001
18. Informing students concerning the result of examinations	3.07	3.79	-0.72	-9.60	<0.001
19. Presenting materials and content understandably	3.26	4.28	-1.01	-15.2	<0.001
20. Gaining higher scores if students attempt more	3.43	4.05	-0.61	-7.90	<0.001
21. Recording students' educational documents without mistake	3.54	3.98	-0.43	-6.10	<0.001
22. Easy accessibility of available references in university	3.45	4.23	-0.78	-11.1	<0.001
23. Fulfilling responsibilities by faculty members and staff in the promised time	3.39	4.05	-0.66	-9.60	<0.001
					
Tangibles					
24. Neat ant professional appearance of faculty members and staff	3.51	3.86	-0.35	-4.70	<0.001
25. Visual appealing and comfort of physical facilities	2.40	3.93	-1.53	-17.8	<0.001
26. Material and educational equipment being up to date	3.33	4.10	-0.77	-10.5	<0.001
27. Visual appealing of teaching tools	3.15	3.86	-0.71	-9.40	<0.001

*Perception **Expectation.

There was no significant difference between perceptions of the students in the Associated degree level, Bachelor of Science (BS) level, and general medicine level, but there were significant differences in expectations among them (Table 3).

**Table 3:** Comparison of the students' perceptions, expectations and service gaps in different educational levels

Service Dimensions	Educational Level	Perception	Expectation	Service gaps
Assurance	Associate degree	3.31	4.06	-0.74
	Bachelor of Science	3.16	4.01	-0.85
	General medicine	3.17	4.40	-1.23
	ANOVA*	p** = 0.15	p < 0.004	p < 0.001
				
Responsiveness	Associate degree	2.80	3.80	-0.99
	Bachelor of Science	2.84	3.90	-1.06
	General medicine	2.68	4.20	-1.52
	ANOVA	p = 0.35	p < 0.005	p < 0.001
				
Empathy	Associate degree	3.13	3.90	-0.76
	Bachelor of Science	2.97	3.95	-0.98
	General medicine	3.06	4.36	-1.30
	ANOVA	p = 0.24	p < 0.001	p < 0.001
				
Reliability	Associate degree	3.44	3.95	-0.51
	Bachelor of Science	3.31	4.06	-0.75
	General medicine	3.28	4.35	-1.07
	ANOVA	p = 0.12	p < 0.002	p < 0.001
				
Tangibles	Associate degree	3.16	3.81	-0.64
	Bachelor of Science	2.98	3.92	-0.94
	General medicine	3.10	4.23	-1.13
	ANOVA	p = 0.26	p < 0.005	p < 0.003
				
Total service quality	Associate degree	3.19	3.91	-0.72
	Bachelor of Science	3.07	3.98	-0.90
	General medicine	3.08	4.32	-1.24
	ANOVA	p = 0.17	p < 0.001	p < 0.001

* Oneway ANOVA **P. value

## Discussion

The aim of this study was to determine the quality gap of educational services using a modified SERVQUAL instrument among students in Hormozgan University of Medical Sciences. As the results show in all of the five SERVQUAL dimensions, there is a negative quality gap. This confirms the results of the Kebriaei and Roudbari [15], Braddley [16], and Clare Chua [17] studies. Negative quality gaps mean students' expectations are greater than their perceptions, and it indicates dissatisfaction. Thus, improvements are needed across all five SERVQUAL dimensions.

In this study, the least and the greatest negative quality gap are in the reliability and responsiveness dimensions respectively. The findings support the results of the Kebriaei and Roudbari study in Zahedan University of Medical Sciences. In a similar study conducted by Ruby, there were negative quality gaps in the reliability, assurance, responsiveness and empathy dimensions, but there was a positive quality gap in the tangibles dimension; in this dimension, students' perceptions of the educational services quality was greater than their expectations [18]. The result of Ruby's study in the tangibles dimension doesn't support the result of this study in this dimension. In the Ruby study, the most negative quality gap was in the reliability dimension, followed by the responsiveness and empathy dimensions, and the least negative quality gap was in the assurance dimension [18]. In the Clare Chua study concerning the educational services quality at Ryerson University in Toronto, the greatest negative quality gap was in the assurance dimension, followed by the responsiveness, tangibles and empathy dimensions, and the least negative quality gap was in the reliability dimension [17].

The negative quality gaps in all of the five SERVQUAL dimensions and their items indicate that in order to improve educational services quality, some measures need to be taken. The greatest negative quality gap was in the responsiveness dimension. This dimension indicates the school's willingness to help students and provide prompt services; it also reflects the sensibility and cautions to students' demands, questions and complaints [17,19]. The greatest negative quality gap in this dimension and its items indicates that supervisors are not accessible when students need them, students don't have easy access to the administrator to express their viewpoints and suggestions regarding the curriculum, students' viewpoints and suggestions are not considered in curriculum, little attention is paid to introducing suitable references to students for reading, and the supervisor's counseling hours are not aptly and properly specified.

Negative quality gaps in other dimensions indicate that responsibilities have not been fulfilled well to meet students' expectations. Given the viewpoints of most students and the negative quality gap in each of the five SERVQUAL dimensions, the following educational workshops are suggested in order to reduce these gaps: "how to communicate with students", "increasing staff skills", and "effective communication of faculty members and students". On the other hand, supervisors should have a schedule for counseling the students and students should be informed of it. Also the administrators should plan working hours of faculty members so that they have enough time for counseling, faculty members should be accessible outside of class to answer students' questions, students should have easy access to the administrator to express their viewpoints and suggestions concerning the curriculum and educational problems, and finally students' viewpoints and suggestions should be considered in curriculum.

In this study there was no significant difference between students' perceptions in Associated degree level, Bachelor of Science (BS) level, and general medicine level, but there was a significant difference between expectations of them. In general, medical students have greater expectations from educational services quality. Also, the negative quality gap in the general medicine level is greater than other educational levels. Thus, in order to reduce the negative quality gap in this level, more attention should be paid to the students' expectations.

## Conclusion

The negative quality gap in service dimensions can be used as a guideline for planning and allocation of resources [20]. Thus, the five SERVQUAL dimensions can be classified to three priority groups for allocation of resources and organizational attempts to eliminate or reduce negative quality gaps, so that the responsiveness dimension is placed in the first priority, the assurance, empathy and tangibles dimensions are placed in the second priority, and the reliability dimension is placed in the third priority. If the afore mentioned priorities are taken into account and the quality gap is attended to, the resultant improved will benefit other dimensions as well; the negative quality gap (or quality improvements) in one dimension, in the customers' viewpoint, can affect the negative quality gaps (or quality improvements) in other dimensions [21].

Due to the diversity of courses and educational levels in other universities and having different facilities, equipment, staff and faculty members, the results of this study are not generalizable to all. Hence it is recommended that every university carry out a similar study so that a model with more conformity will be produced for planning to improve educational services quality.

## Competing interests

The authors declare that they have no competing interests.

## Authors' contributions

TA designed and conducted the study, drafted and edited the manuscript. SZ designed the methodology and analyzed the data.

## Pre-publication history

The pre-publication history for this paper can be accessed here:


